# Baltimore College of Dental Surgery

**Published:** 1891-04

**Authors:** 


BALTIMORE COLLEGE OF DENTAL SURGERY.—The fifty-first annual Commencement exercises of the Baltimore College of Dental Surgery, Prof. R. B. Winder, Dean, were held at Harris's Academy of Music, Baltimore, Md., on Monday evening, March 23, 1891.

The annual oration was delivered by Dr. Wayland Ball, and the valedictory address by Edward Hamm, D. D. S., of the graduation class.

The number of graduates was seventy-six, and the number of matriculation for the session was two hundred and twenty-four.

The degree of D. D. S. was conferred on the following graduates by Professor M. W. Foster:

David L. Aber, Pennsylvania; Charles E. Altemus, Pennsylvania; Louis D. Archinard, Louisiana; Walter V. Austin, Minnesota; Theodore A. Bailey, Georgia; Calvin D. Brown, California; John H. Bulett, Pennsylvania; James K. Burgess, Virginia; James F. Butts, West Virginia; Joseph J. Battle, North Carolina; Samuel E. Beecher, Pennsylvania; Horace I. Beamer, New Jersey; Ernest Bent, Massachusetts; Robert C. Bradshaw, Maryland; John J. Carroll, West Virginia; Frank M. Conkey, Illinois; Willard L. Chapin, Pennsylvania; Calvin O. Chunn, Florida; L. M. Cleckley, Georgia; Charles G. Colby, Connecticut; Thomas A. Cronin, Maryland; Ulysses A. Dalton, New York; John W. David, Texas; Ernest C. Deuel, California; James F. Downs, Maryland; William Dick, California; Edward Eggleston, Virginia; Josiah G. Fife, Texas; James G. Findlay, Ontario; Branch Garner, Louisiana; James B. Gould, Virginia; William F. Graham, South Carolina; Harvey T. Greenlaw, New Brunswick; Guy Gress, Pennsylvania; Edward Hamm, Massachusetts, Lewis A. Hauser, North Carolina; Samuel J. Heindel, Pennsylvania; Elmer E. Henry, Pennsylvania; Robert S. Henry, Maryland; Frank W. Hill, Iowa; George H. Jackson, Pennsylvania; Walter L. Jones, Mississippi; George Kress, Ohio; T. B. Leatherbury, Virginia; Harold S. Lockwood, New York; John C. Maloney, Virginia; E. B. Marshall, Jr., Georgia; G. V. Milholland, Maryland; Frank S. Morton, Nova Scotia; James S. McDonald, Pennsylvania; William E. Nye, California; Cameron E. Orndorf, Pennsylvania; Edward B. Parker, Virginia; Oswald A. Parker, Nova Scotia; John C. Pfeiffer, Maryland; Mozart W. Rainold, Louisiana; Hermann Reichhelm, Germany; Charles P. Rice, Pennsylvania; George O. Roberts, Germany; Fred. C. Royce, New York; G. P. Schumacker, Massachusetts; Thomas W. Sharpe, Pennsylvania; Warren M. Sharp, New Brunswick; Otis F. Sims, Florida; W. M. Steinmeyer, South Carolina; Wm. M. Stewart, New York; George E. Stoddard, Vermont; Charles B. Tarr, Maine; W. E. Teasley, Virginia; Thomas K. Tharp, Georgia; Rowland H. Walker, Virginia; Frederick R. Wilder, Vermont; Frank C. Wilson, Georgia; Hermann Wurzel, Germany; W. E. Wolfrum, Wisconsin; Andrew Youngs, New York.

